# Abnormal regional homogeneity in right caudate as a potential neuroimaging biomarker for mild cognitive impairment: A resting-state fMRI study and support vector machine analysis

**DOI:** 10.3389/fnagi.2022.979183

**Published:** 2022-09-01

**Authors:** Yujun Gao, Xinfu Zhao, JiChao Huang, Sanwang Wang, Xuan Chen, Mingzhe Li, Fengjiao Sun, Gaohua Wang, Yi Zhong

**Affiliations:** ^1^Department of Psychiatry, Renmin Hospital of Wuhan University, Wuhan, China; ^2^Wuxi Mental Health Center, Nanjing Medical University, Wuxi, China; ^3^Affiliated Shuyang Hospital, Nanjing University of Chinese Medicine, Suqian, China; ^4^Department of Psychiatry, The First Affiliated Hospital of Xinxiang Medical University, Xinxiang, China; ^5^Peking-Tsinghua Center for Life Sciences and PKU-IDG/McGovern Institute for Brain Research, Peking University, Beijing, China; ^6^Medical Research Center, Binzhou Medical University Hospital, Binzhou, China; ^7^Department of Neuroscience, City University of Hong Kong, Kowloon, Hong Kong SAR, China; ^8^NHC Key Laboratory of Mental Health, Peking University Sixth Hospital, Peking University Institute of Mental Health, Peking University, Beijing, China

**Keywords:** mild cognitive impairment, neuroimaging, fMRI, regional homogeneity, support vector machine, machine learning, biomarker

## Abstract

**Objective:**

Mild cognitive impairment (MCI) is a heterogeneous syndrome characterized by cognitive impairment on neurocognitive tests but accompanied by relatively intact daily activities. Due to high variation and no objective methods for diagnosing and treating MCI, guidance on neuroimaging is needed. The study has explored the neuroimaging biomarkers using the support vector machine (SVM) method to predict MCI.

**Methods:**

In total, 53 patients with MCI and 68 healthy controls were involved in scanning resting-state functional magnetic resonance imaging (rs-fMRI). Neurocognitive testing and Structured Clinical Interview, such as Alzheimer's Disease Assessment Scale-Cognitive Subscale (ADAS-Cog) test, Activity of Daily Living (ADL) Scale, Hachinski Ischemic Score (HIS), Clinical Dementia Rating (CDR), Montreal Cognitive Assessment (MoCA), and Hamilton Rating Scale for Depression (HRSD), were utilized to assess participants' cognitive state. Neuroimaging data were analyzed with the regional homogeneity (ReHo) and SVM methods.

**Results:**

Compared with healthy comparisons (HCs), ReHo of patients with MCI was decreased in the right caudate. In addition, the SVM classification achieved an overall accuracy of 68.6%, sensitivity of 62.26%, and specificity of 58.82%.

**Conclusion:**

The results suggest that abnormal neural activity in the right cerebrum may play a vital role in the pathophysiological process of MCI. Moreover, the ReHo in the right caudate may serve as a neuroimaging biomarker for MCI, which can provide objective guidance on diagnosing and managing MCI in the future.

## Introduction

Mild cognitive impairment (MCI) is a heterogeneous syndrome defined as deficits on neurocognitive testing but without significant damage to activities of daily living (ADLs) (Winblad et al., 2004). Objective evidence cognitive or functional decline, labeled “subjective cognitive decline,” does not always accompany subjective awareness of cognitive impairment (Tao et al., 2021). MCI reflects that cognitive function deteriorates in neuropsychological testing, but ADL is intact relatively (Petersen, 2006).

One characteristic of MCI is heterogeneous, which means that about 5–15% of the patients are at the risk of getting dementia every year. At the same time, about half of the patients keep stable at 5 years, and symptoms sort out over time in the minority (Dunne et al., 2021). The collaboration of Cohort Studies of Memory in an International Consortium (COSMIC), which spread standard diagnostic criteria to estimate the prevalence of MCI more reliably across different regions, confirmed the prevalence of MCI in adults over 60 years up to 6% across 11 studies (Sachdev et al., 2015).

The guideline date updated by the American Academy of Neurology estimated a prevalence of 6.7% in 65–69-year-olds and 25% for 80–84-year-olds (Livingston et al., 2017). Jia et al. estimated that overall MCI prevalence was 15.5% in China, representing 38.77 million people (Jia et al., 2020). Consistently, 10 million new instances of dementia are enlisted, and it is assessed that 13,500,000 individuals are associated with the risk of dementia by 2050 (WHO., 2022). It presents an opportunity for reducing vascular risk and altering behavioral patterns when a diagnosis of MCI is made. Given diagnostic and therapeutic practice variation, things could change if a more definitive diagnosis is available. Therefore, standard national guidance is eagerly needed for using neuroimaging in MCI (Gillis et al., 2019; McWhirter et al., 2020). There is a need for precise prognosis methods to ensure that the diagnosis of MCI is a possibility for people to prevent the risk of Alzheimer's disease (AD). To improve the specificity and sensitivity of MCI diagnosis, we need cognitive testing and neuroimaging with more evidence.

Resting-state functional magnetic resonance imaging (rs-fMRI) is a non-invasive imaging tool to detect brain activity (Biswal et al., 1995; Smitha et al., 2017). As shown in previous research, rs-fMRI has been confirmed as a reliable instrument to explore the brain's mechanism, reflecting the signature of the brain's neural network (van den Heuvel and Hulshoff Pol, 2010; Pan et al., 2021). Regional homogeneity (ReHo) is a test-retest method to detect the characteristic of brain connectome (Biswal, 2012; Cheng et al., 2019; Geng et al., 2019; Hou et al., 2021). The support vector machine (SVM) is among the most popular methods in the machine learning (ML) that has exceeded practical neuroimage analysis in the past 20 years (Orru et al., 2012; Pisner and Schnyer, 2020). SVM has been applied in different fields and addresses various classification problems because of its relative simplicity and flexibility (Gori et al., 2015; Cheng et al., 2021; Shan et al., 2021; Gao et al., 2022). There have been hundreds of studies using ML to accurately classify patients with heterogeneous mental and neurodegenerative disorders, which make improvements in the early diagnosis.

In brain disorders research, SVMs are deployed by multivoxel pattern analysis (MVPA) and solve plenty of clinical problems because of their relative simplicity. Even if data are high dimensional, there is a lower risk of overfitting in SVMs (Zhang et al., 2020). Recently, SVMs have been applied in precision medicine. SVM analysis can predict the diagnosis and prognosis, particularly for patients with brain diseases, such as Alzheimer's (Franzmeier et al., 2020; Xie et al., 2022), schizophrenia's (Chand et al., 2022; Lei et al., 2022), and depression's (Al-Hakeim et al., 2019; Bone et al., 2021). The results would make improvement in the diagnostic accuracy.

More research participation is required to increase the investment in neuroimaging biomarkers to achieve this goal. To determine whether there are distinct or specific alterations in MCI that can be used to differentiate MCI from healthy controls, we examined specific or distinctive alterations in MCI. The study hypothesized that patients with MCI have abnormal ReHo in the brain region and the abnormal region serves as a biomarker to aid in diagnosing and prediction of MCI.

## Methods

### Participants and procedures

In total, 140 subjects were consecutively recruited from Wuxi Mental Health Center in China between June 2018 and May 2022.

In total, 70 patients with MCI and 70 age-, education-, and gender-matched healthy subjects, which were right-handed, were recruited. The patients were diagnosed by two psychiatrists with criteria in the Structured Clinical Interview of the fifth version of the Diagnostic and Statistical Manual of Mental Disorders (DSM-V) independently. All participants were assessed by Alzheimer's Disease Assessment Scale-Cognitive Subscale (ADAS-Cog) test (Kueper et al., 2018), ADL Scale, Hachinski Ischemic Score (HIS), Hamilton Rating Scale for Depression-17 (HRSD), Clinical Dementia Rating (CDR), and Montreal Cognitive Assessment (MoCA). The patients who met the criteria (CDR ≥ 0.5, MoCA <26) were included. Exclusion criteria were as follows: history of brain damage, other neurological disorders, other mental disorders, such as acute illness, substance abuse, schizophrenia, dementia, bipolar disorder, and other severe limb or head tremor. All healthy comparisons (HCs) were recruited from the community as volunteers without a history of neurologic disorders. In control comparisons, neuropsychology evaluations were also performed the same as patients with MCI. The study was approved by the Medical Ethics Committee of Wuxi Mental Health Center. Each participant had written informed consent and submitted it before enrollment.

### Materials

#### ADAS-Cog test

The ADAS-Cog helps to evaluate cognition and differentiation between normal and impaired cognitive functioning. The administrator accumulates points for the errors in the test task for a total score. The score ranges from 0 to 70 and a score of 70 epitomizes the most severe impairment (Cano et al., 2010).

#### The ADL scale

The ADL is used to describe fundamental skills in taking care of oneself independently (Edemekong et al., 2022). ADL is used as an indicator of a person's functional status and to assess older adults' functional status (Árnadóttir, 2016).

#### Hachinski ischemic score

The vascular burden is considered a risk factor for cognitive dysfunction. HIS is a clinical tool used to identify vascular dementia (Johnson et al., 2014). The scores were related to cognitive functioning, such as global cognition, executive functioning, immediate memory, and attention. HIS can significantly predict the diagnosis of MCI (Paul et al., 2003).

#### Hamilton rating scale for depression

A 17-item HRSD was used to assess the symptom of depression (Hamilton, 1967).

#### Clinical dementia rating

Clinical Dementia Rating is a global rating device calculated on six behavioral and cognitive fields, such as memory, orientation, problem solving, judgment, personal care, and home and hobbies performance. The CDR is based on a scale ranging from 0 to 3: no dementia (CDR = 0), questionable dementia (CDR = 0.5), MCI (CDR = 1), moderate cognitive impairment (CDR = 2), and severe cognitive impairment (CDR = 3) (Khan, 2016).

#### Montreal cognitive assessment

Montreal Cognitive Assessment score ranges from 0 to 30 points, and a cut score of 26 represents excellent specificity and sensitivity separating MCI from healthy subjects (De Reuck, 2020).

### Image acquisition

All the rs-MRI data were obtained by an Achieva 3.0T Scanner (Philips, Amsterdam, the Netherlands) at the Wuxi Mental Health Center on the first day. Participants were instructed to remain still, close their eyes, and avoid falling asleep during the scan. Echo-planar imaging of resting-state functional images was performed using the following parameters: repetition time/echo time (TR/TE) 2,000/30 ms, 31 slices, 90° flip angles, 22 cm × 22 cm field of view (FOV), 5 mm slice thickness, and 1 mm pitch.

### Data preprocessing

Imaging data were preprocessed with Data Processing Assistant for Resting-State (DPARSF) software in MATLAB (Yan et al., 2016). It was necessary to exclude the first five time points to minimize the error caused by the initial signal instability and participants' adaptation times. Following this, time-slice correction and head movement correction were completed. Maximum displacements in x-, y-, or z-axes and maximum rotations in angular direction were no more than 2 mm and 2°, respectively. Image data were corrected for spatial normalization to the standard Montreal Neurological Institute (MNI) space. Samples were resampled to 3 × 3 × 3 mm. Filter was used along with linear detrending (0.01–0.08 Hz) and linearly detrended. A series of covariates were removed that included the signal from a region centered in the white matter, and six head-motion parameters were calculated from rigid bodies (Gao et al., 2022).

### ReHo analyses

Using the Kendall coefficient of concordance (KCC) between the time series of a given voxel and the 26 nearest voxels, we generated the ReHo map for each participant. The non-brain tissues were then removed using a whole-brain mask. As part of the standardization process, we divided each ReHo map by its global mean KCC within the whole-brain mask. Spatial smoothing of the ReHo maps was performed with a Gaussian kernel of 4.5 mm full-width at half-maximum (FWHM).

### Statistical analysis

With the help of two-sample *t*-tests, age, education, and whole-brain voxel-based maps were compared. The gender was compared by the chi-square test. By controlling topological family-wise errors (FWEs) computed using Gaussian Random Field theory, all significant clusters were corrected at the cluster level. The threshold was *p* < 0.01 for cluster-forming voxel-level heights, and the threshold was *p* < 0.05 for cluster-wise FWEs. Gray matter (GM) masks were generated group wise, i.e., voxels.

### Classification analysis

For each participant, we calculated the Pearson correlation between the time series of all pairs of brain voxels. In MATLAB, LIBSVM was used to run the SVM method. With ReHo values extracted from different brain regions, SVM was applied to test the ability to distinguish patients from healthy controls.

## Results

### Demographics

From June 2018 to May 2022, 140 participants were enrolled. Among all participants, 17 patients and 2 HCs were excluded because of the head motion parameters. The remaining 53 patients with MCI and 68 age-, education-, and sex-matched control volunteers were recruited. The subjects were divided into 2 groups: patients with MCI (n = 53) and HCs (n = 68). Demographic information and clinical assessment scores are shown in Table 1. There were no significant differences in age, gender, and year of education between the two groups (*p* > 0.05). Compared with the HCs, MCI patients has poorer scores in all cognition tests, including ADAS-Cog test (*p* < 0.05), HIS (*p* < 0.05), CDR (*p* < 0.05), and MoCA (*p* < 0.05). There was no significance in the ADL Scale and HRSD between groups (*p* > 0.05, Table 1).

**Table 1:** Demographics of the study population.

	**MCI (*n =* 53)**	**Control (*n =* 68)**	***P***
Age(years)	69.07 ± 4.932	68.80 ± 4.951	*P* > 0.05
Sex(M/F)	40/30	34/34	*P* > 0.05
Education (years)	10.04 ± 3.321	9.75 ± 3.414	*P* > 0.05
ADL	14.243 ± 0.824	14.059 ± 0.237	*P* > 0.05
HRSD	2.114 ± 2.446	1.441 ± 2.153	*P* > 0.05
ADAS-Cog	9.527 ± 3.480	7.066 ± 2.760	*P* < 0.05
HIS	1.371 ± 0.819	1.044 ± 0.818	*P* < 0.05
CDR	0.814 ± 0.889	0.316 ± 0.487	*P* < 0.05
MoCA	22.814 ± 4.150	26.265 ± 1.671	*P* < 0.05

ADAS-Cog, Alzheimer's Disease Assessment Scale-Cognitive Subscale test; ADL, Activity of Daily Living Scale; HIS, Hachinski ischemic Score; CDR, Clinical Dementia Rating; MoCA, Montreal Cognitive Assessment; HRSD, Hamilton Rating Scale for Depression. The *p*-value was obtained by *t*-tests and gender was compared by Chi-square test.

### ReHO: Patients vs. HCs

**Figure 1:**
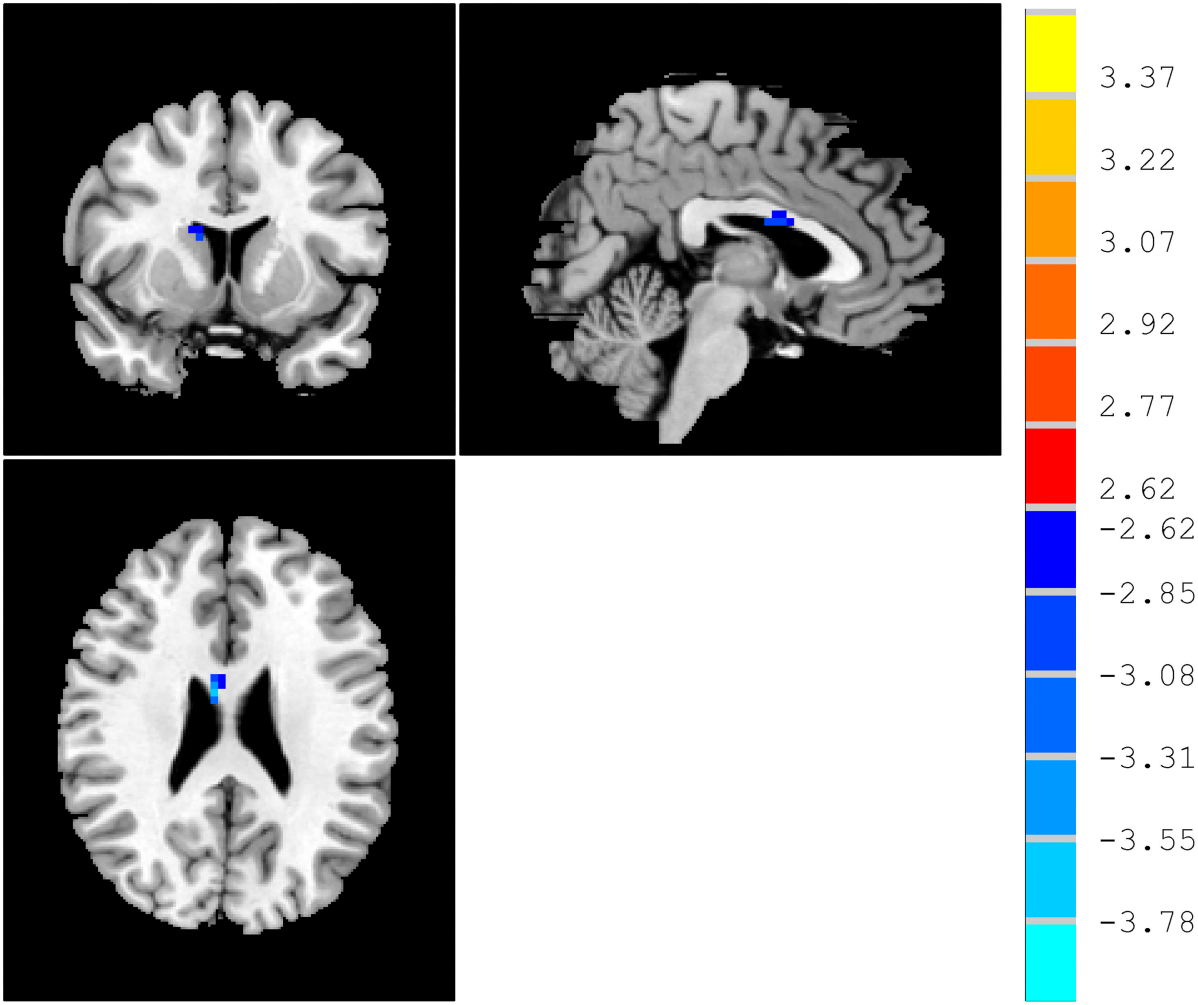
ReHo differences between patients with MCI and HCs. Red and blue denote higher and lower ReHo, respectively, and the color bars represent the *t-*values from the *t*-test of the group analysis. ReHo, regional homogeneity; MCI, mild cognitive impairment; HCs, healthy comparisons.

Compared with HCs, patients with MCI displayed a significantly decreased ReHo in the right caudate (Table 2 and Figure 1).

**Table 2:** ReHo difference among MCI and healthy subjects.

**Brain areas (AAL)**	**Peak MNI coordinates**			**Cluster size**	**Peak *T* value**
	**X**	**Y**	**Z**	
MCI vs. CONTROL Right caudate	6	−3	21	43	−4.0112

ReHo, regional homogeneity; MCI, mild cognitive impairment; MNI, Montreal Neurological Institute.

### The results of the SVM

**Figure 2:**
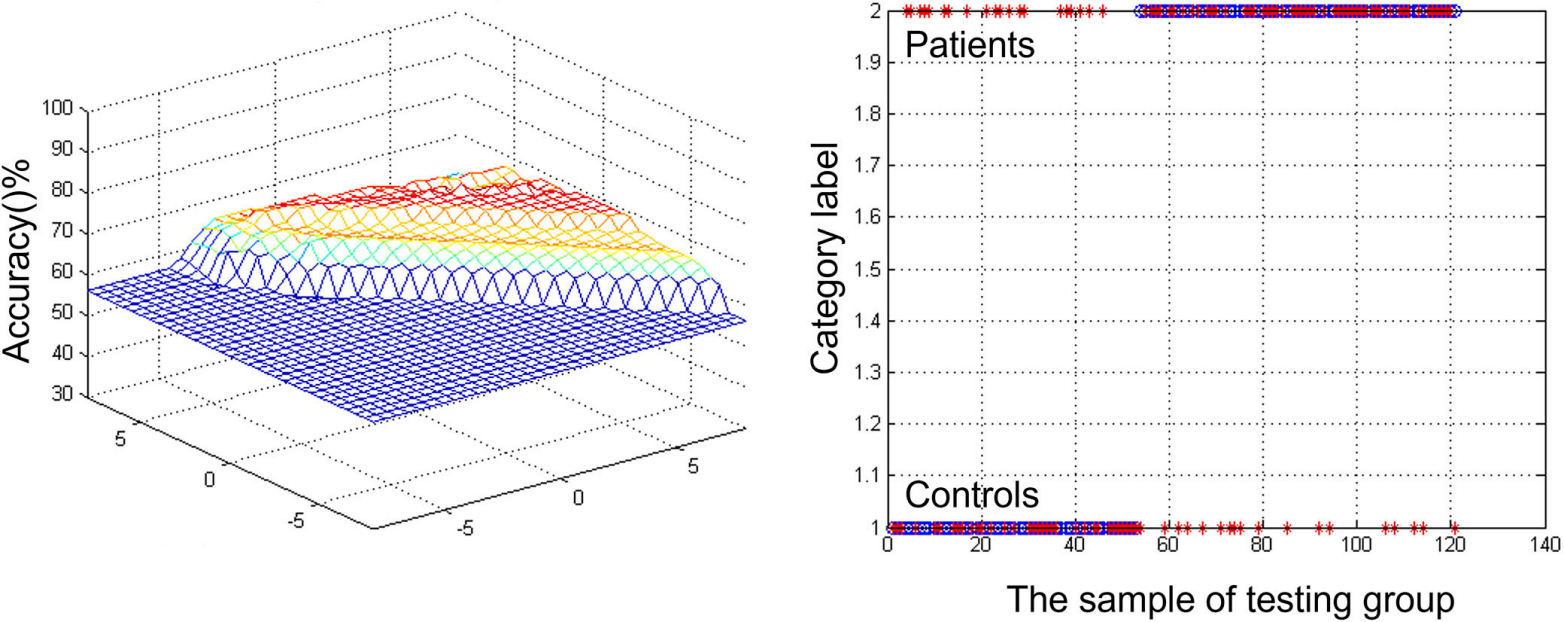
Visualizing classifications based on support vector machine (SVM) by the decreased regional homogeneity (ReHo) values in the right caudate to discriminate patients with mild cognitive impairment (MCI) from healthy comparisons. **(Left)** SVM parameters' result of 3D view. **(Right)** Classified map of the ReHo values in the right caudate.

A combination of decreased ReHo in right caudate was a potential biomarker to diagnose MCI by SVM. The SVM classification achieved an overall accuracy (68.6%), sensitivity (62.26%), and specificity (58.82%) (Figure 2).

## Discussion

Mild cognitive impairment could be a prodromal stage of dementia. Over the past decade, neuroimaging has gained increasing attention as a predictor of mental disorders (Grueso and Viejo-Sobera, 2021). The association between MCI and right caudate was assessed in fMRI comprising 53 MCI patients with a range of demographic characteristics, and HCs were matched to patients. As shown in the results, when compared with the HCs, patients with MCI had poorer scores in all cognition tests, such as ADAS-Cog test (*p* < 0.05), HIS (*p* < 0.05), CDR (*p* < 0.05), and MoCA (*p* < 0.05). There was no significance in the ADL Scale and HRSD between the two groups (*p* > 0.05). Patients with MCI at times will ultimately advance to dementia, yet the cognitive symptoms have not ultimately shown.

Compared with HCs, the ReHo of patients with MCI in the right caudate is lower than HCs. The caudate nucleus plays a critical role in various higher neurological functions. It is a region that is not only in executive functioning but also related to learning, memory, motivation, and emotion (Fisher et al., 2006; Grahn et al., 2008). One can consider the head of the caudate nucleus as the cognitive and emotional portion (Seger and Cincotta, 2005; Graff-Radford et al., 2017). It is possible that the caudate was restricted from overactivity due to its significant involvement in the working memory task. Ekman et al. found in patients with Parkinson's disease and MCI, blood-oxygen-level-dependent (BOLD) signals are reduced in the right caudate and frontal cortex, as well as impaired presynaptic function in the caudate (Ekman et al., 2012). Working memory is updated by the caudate and anterior cingulate cortex and their functional changes are associated with Parkinson's disease with MCI. Specifically, Qiu et al. found that MCI smokers showed decreased functional connectivity of right caudate to left inferior parietal lobule (Qiu et al., 2022). Compared to controls, caudate volumes were lower in MCI (4.43% right) (Madsen et al., 2010).

Nevertheless, in the early stages, although biomarkers might be found in the result of fMRI, it stays trying for the location of MCI to dementia progression in clinical practice (Zhang et al., 2012). For this reason, finding the contrast between those patients with MCI and healthy subjects is significant. To tackle this issue, researchers established neuroimaging datasets from patients with MCI, HCs, and different variables, such as demographic, genetic, and cognitive measurements (Pellegrini et al., 2018). SVM can characterize non-linear choice limits in high-layered variable space by tackling a quadratic improvement issue (Grueso and Viejo-Sobera, 2021). This technique can provide a valuable insight into a disease that occurs with neural patterns, such as autism (Gori et al., 2015), attention deficit hyperactivity disorder (Park et al., 2016), and schizophrenia (Cheng et al., 2015; Pina-Camacho et al., 2015; Arbabshirani et al., 2017). In the study, the decreased ReHo of right caudate was a potential biomarker to diagnose MCI by SVM. The SVM results showed a diagnostic accuracy of 68.6% (83/121). The ReHo was decreased in the right caudate, with a sensitivity of 62.26% (33/53) and a specificity of 58.82% (40/68). The level of prediction may seem modest, but it must be viewed in the context of other risk factors that patients may have for progression over the next 20–30 years. Since these patients usually coexist, it is crucial to understand how they interact. The results suggest that future therapy studies should directly verify the ability to deliver compounds to the caudate nucleus as the cognitive and emotional portion.

In summary, this study is the first to demonstrate the relation between the right cerebrum and MCI using ML based on fMRI imaging data. The right cerebrum may play an important role in early intervention in patients with MCI. It would be interesting to combine ReHo value and MCI to fully investigate its diagnostic accuracy.

They can serve as an objective and reliable complementary tool to improve diagnosis accuracy and ultimately predict the MCI. In addition, by classifying biomarkers, it is possible to revise clinical diagnosis in cases of uncertain diagnosis. The results add to a superior comprehension of the mechanism in the cognitive decline of patients with MCI.

## Limitation

With regard to the research methods, some limitations need to be acknowledged. First, cross-sectional design limits our ability to interpret causal relationships between patients with MCI and HCs. This study needs to be confirmed in the future to increase the likelihood of generalizing its results to MCI patients with different clinical characteristics. Second, several factors confound our study, such as illness duration, medication, lifestyle, and diet. In the future, we will explore potential variations. Third, there was no information available about whether changes occurred in the caudate before the development of MCI. Understanding the cause and effect may be possible through long-term follow-up observations. Fourth, we do not make the use of the medial temporal lobe atrophy (MTA) score (Scheltens' scale) in distinguishing patients with MCI and AD from those without impairment.

## Conclusion

In conclusion, the results suggest that abnormal neural activity in the right cerebrum plays a vital role in the process of MCI. Moreover, the ReHo in right caudate may serve as a neuroimaging biomarker for MCI, which can provide objective guidance on diagnosing and managing MCI in the future.

## Data availability statement

The raw data supporting the conclusions of this article will be made available by the authors, without undue reservation.

## Ethics statement

The studies involving human participants were reviewed and approved by Medical Ethics Committee of Wuxi Mental Health Center. The patients/participants provided their written informed consent to participate in this study. Written informed consent was obtained from the individual(s) for the publication of any potentially identifiable images or data included in this article.

## Author contributions

GW, YZ, and FS: conception and design. YG: statistical analysis. YZ: drafting of the manuscript. XZ, JH, and SW: conduction. XC and ML: critical revision of the manuscript. All authors read and approved the final paper.

## Conflict of interest

The authors declare that the research was conducted in the absence of any commercial or financial relationships that could be construed as a potential conflict of interest.

## Publisher's note

All claims expressed in this article are solely those of the authors and do not necessarily represent those of their affiliated organizations, or those of the publisher, the editors and the reviewers. Any product that may be evaluated in this article, or claim that may be made by its manufacturer, is not guaranteed or endorsed by the publisher.
